# *msemalign*: a pipeline for serial section multibeam scanning electron microscopy volume alignment

**DOI:** 10.3389/fnins.2023.1281098

**Published:** 2023-12-12

**Authors:** Paul V. Watkins, Eric Jelli, Kevin L. Briggman

**Affiliations:** Max Planck Institute for Neurobiology of Behavior—caesar, Bonn, Germany

**Keywords:** multibeam, alignment, serial section electron microscopy, pipeline, SEM

## Abstract

Serial section multibeam scanning electron microscopy (ssmSEM) is currently among the fastest technologies available for acquiring 3D anatomical data spanning relatively large neural tissue volumes, on the order of 1 mm^3^ or larger, at a resolution sufficient to resolve the fine detail of neuronal morphologies and synapses. These petabyte-scale volumes can be analyzed to create connectomes, datasets that contain detailed anatomical information including synaptic connectivity, neuronal morphologies and distributions of cellular organelles. The mSEM acquisition process creates hundreds of millions of individual image tiles for a single cubic-millimeter-sized dataset and these tiles must be aligned to create 3D volumes. Here we introduce *msemalign*, an alignment pipeline that strives for scalability and design simplicity. The pipeline can align petabyte-scale datasets such that they contain smooth transitions as the dataset is navigated in all directions, but critically that does so in a fashion that minimizes the overall magnitude of section distortions relative to the originally acquired micrographs.

## Introduction

Over the last decades, interest in the collection of ever larger EM volumes for connectomics research has grown. In order to reach petabyte-scale datasets with reasonable acquisition durations, some form of parallelized acquisition is required. Serial section multibeam scanning electron microscopy (ssmSEM) is among the fastest technologies currently available for collecting cellular and synaptic resolution anatomy data of relatively large tissue volumes. Our approach is to use a customized sectioning technique (Fulton et al., 2023) along with parallel EM micrograph acquisition using the Zeiss multiSEM (Eberle et al., 2015). Such mSEMs image by scanning multiple beams in parallel over slightly overlapping regions, meaning that 2D sections are subdivided into many small image tiles (each 15.5 × 13.5 μm^2^). As an example, using these techniques to create an EM volume from a cubic mm of tissue cut with 35 nm section thickness and imaged at 5 nm pixel size results in about 1 petabyte of data, which consists of on the order of 100 million individual 2D-overlapping image tiles. Assembling a cohesive volumetric dataset from this quantity of individual image tiles requires computationally aligning them with respect to each other. Such an alignment is a computational challenge because: (1) section deformations that occur during cutting and placement on a wafer are non-linear, (2) artifacts (debris, charging artifacts, holes, etc…) and missed sections can occur during the sectioning and acquisition, and (3) the shear amount of data involved requires efficient algorithms and scalability to parallelized hardware resources. In particular it is important that the alignment of EM datasets can proceed at a similar speed (or faster) relative to the acquisition of the raw data, so that it does not become the major bottleneck for EM-based connectomics. For the aforementioned reasons, several alignment packages and pipeline designs have been developed in recent years.

Here we describe *msemalign*, a software package and pipeline for aligning ssmSEM tissue volumes. At the time that our development on *msemalign* began, our pipeline drew inspiration in particular from two existing approaches (Schaefer et al., 2006; Saalfeld et al., 2012), for which we initially encountered some problems adapting to our acquisition format and scaling. Thus, we created *msemalign*, a pipeline mostly designed with classic image processing techniques, focused on aligning and correcting artifacts based on the specifics of our sectioning and acquisition methodologies and designed to be scalable using high performance computing (HPC) resources up to petabyte-scale datasets. Existing state-of-the-art aligners have been designed with an emphasis on modularity and scalability (Vescovi et al., 2020; Mahalingam et al., 2022), which is important for scaling to even larger datasets. However, for labs with reasonable but modest HPC resources, financing and/or implementing and maintaining such multifaceted storage and database systems may be prohibitive. Therefore we took inspiration from approaches using a combination of image-feature based (Saalfeld et al., 2010, 2012; Khairy et al., 2018; Mahalingam et al., 2022) and cross correlation based (Bock et al., 2011; Briggman et al., 2011; Scheffer et al., 2013) image registration techniques. Although *msemalign* is less comprehensive than existing toolsets designed for more general bioimaging applicability (Cardona et al., 2012), it is kept lean and dependency-light by focusing on alignments for serial sections acquired with GAUSS-EM (Fulton et al., 2023) and imaged with a mSEM. Again with an emphasis on simplicity, we opted for a solution which employs robust outlier detection. This allows us to employ a series of linear system solvers, based on large systems of coordinate differences, as opposed to solutions involving non-linear solvers (Saalfeld et al., 2012), machine learning (Macrina et al., 2021), or frequency-domain registration (Wetzel et al., 2016) and still obtain good alignments.

At least three other approaches have been described for aligning petascale serial-section EM datasets (Vescovi et al., 2020; Macrina et al., 2021; Mahalingam et al., 2022). These solutions are modular, with components and alignment information being communicated between components via database solutions. We have focused instead on simplicity, i.e., no database requirements, but instead simply serializing alignment information and writing iteratively refined aligned stacks to disk. Most of *msemalign* utilizes traditional image processing techniques, thus simplifying the compute time and oversight of training paradigms typically required by machine-learning-based approaches (Macrina et al., 2021; Mahalingam et al., 2022). We have also focused heavily on improvement of our ssmSEM acquisition, resulting in datasets with greatly reduced incidence of cracks (tears) and folds and of partial sections. Thus, any sections that are heavily deformed, in our case a small percentage of sections containing tears, can be corrected using a semi-automated technique. This also allows for a simplification of the alignment relative to approaches required for datasets containing many cracks or folds on practically every section (Macrina et al., 2021).

A well-aligned volume comprises two major features: (1) continuity of tissue structures (membranes, organelles, etc.) in all directions, and (2) minimal deformation of the original section images. Often only the first item is considered as being important, for example, one of the most common methods to qualitatively assess alignment quality is by examining XZ or YZ reslices of aligned volume data. Although this is important, it is not the only consideration, because without constraining the magnitude of warping to be applied, it is possible to create an aligned volume that has very smooth transitions as the dataset is navigated in any direction, but that also contains unrealistic warping of the original raw data. Thus, a good alignment is one that meets the traditional definition of alignment, so that structures can easily be traced in the volume, but that does so by applying the minimal amount of distortions to the acquired images. Overall *msemalign* is a heavily constrained but fully elastic aligner. This allows tissue sections to be warped non-linearly, in order to recover from deformations that occur during sectioning, but prevents over-warping that may heavily alter morphologies relative to their state in the original tissue volume. All code required for running and scaling the pipeline is publicly accessible, including the main component package *msemalign*.

## Materials and methods

### Aligned volumes

The zebrafish retina dataset was sectioned with 35 nm thickness into 2,592 sections and collected onto three silicon wafers. A total of 19 sections were excluded due to more severe artifacts, so the aligned dataset contains 78,750 × 75,344 × 2,573 voxels. The final volume crops some perimeter from the originally imaged micrographs, which include bare resin, iron/resin mixture or silicon wafer areas. The aligned volume is publically available.^1^ We have also used the *msemalign* package to align two larger ssmSEM datasets with final aligned sizes of 370 TB (a 1 × 1.1 × 0.2 mm volume comprising 5,714 sections) and 378 TB (a 0.75 × 1.2 × 0.26 mm volume comprising 7,495 sections).

### Runtimes

Our compute cluster at the time of alignment contained two partitions, one CPU-only partition containing 26 nodes with 40 cores each (1,040 CPU cores) and one CPU-GPU hybrid partition containing 28 nodes with one NVIDIA 2080Ti GPU and 20 cores (560 CPU cores) each. Using these resources and excluding the time involved in computationally solving the section ordering, the entire pipeline for aligning the zebrafish retina dataset ran in 109 h. We have included a detailed breakdown of the workflow steps as the percentage contribution to the total runtime (Table 1). We have upgraded to a new cluster containing three partitions, two CPU-only partitions, one containing 80 nodes with 48 cores each (3,840 CPU cores) and one containing 24 nodes with 72 cores each (1,728 CPU cores) and one CPU-GPU hybrid partition containing 84 nodes with four NVIDIA Quadro RTX 6000 GPUs and 48 cores (4,032 CPU cores) each. Extrapolating the runtime on the older smaller cluster by directly scaling by the percentage of pixels in zebrafish retina dataset (1,000 TB / 14 TB), the runtime on a 1 PB dataset would be approximately 47 days. Because the CPU/GPUs are more modern, and IO/memory bandwidth and size are also larger on the newer cluster, this estimate represents an upper bound. The primary bottlenecks of the *msemalign* pipeline currently are the computation of normalized cross-correlations for the 3D fine and ultrafine alignments, the many Random Sample Consensus (RANSAC) iterations required for robust 2D vector field outlier detection and the final export at 4 nm. The major parameters that can be adjusted in order to reduce run time for the 3D cross correlations and outlier detection steps are the spacing of the grid points, the number of neighbors, the number of template angles and the size of the source and destination crops. These steps, in particular utilizing more output blocks for the final export at 4 nm, are easily further scalable with more hardware resources. Ultimately the primary bottleneck for the 4 nm export steps is disk IO because aligned stacks currently must be written to disk after the region (2D), fine and ultrafine alignment steps.

**Table 1:** Table showing the percentage of the total runtime that is required for each step.

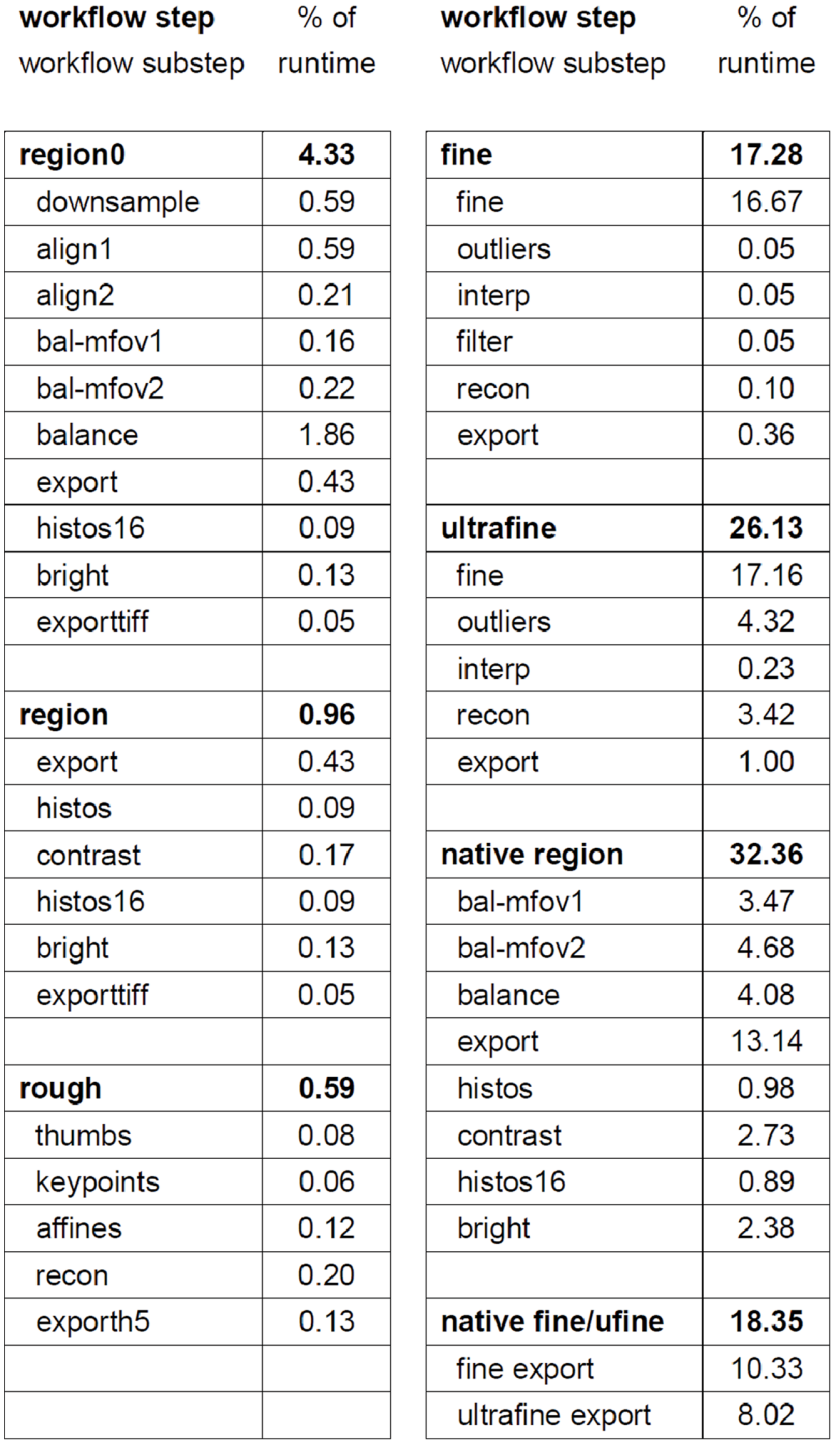

Steps are shown in bold along with corresponding percent contributions to the total runtime. Steps are broken down into substeps (not-bolded), which are also shown as the percentage of total runtime for the whole pipeline. The substep names share those of the *msemalign* package pipeline files that are used to automate the workflow submission to a compute cluster.

### Code availability

The *msemalign* package^2^ that implements the pipeline is released along with two associated packages: *swarm-scripts*^3^ and *rcc-xcorr*.^4^ Most of the parallelization of the pipeline is done without communication, i.e., separate python processes work on different portions of the dataset in each step and do not require inter-node communication. The *swarm-scripts* package is a collection of scripts that can be used along with the *msemalign* pipeline as a most basic method of scaling onto a cluster that uses SLURM scheduling software, by essentially specifying different command lines for each step with parameters that indicate what part of the data that the current script should process. The majority of this parallelization is done by processing sections in parallel, with some portions also parallelized blockwise per section. The *msemalign* and *rcc-xcorr* packages are implemented using scientific Python. Most components of the pipeline also include per-node parallelization, accomplished using Python multiprocessing.

### Normalized cross correlations

Both the *msemalign* and *rcc-xcorr* packages contain routines for computing the normalized cross-correlations (nxcorr) that are utilized during both the 2D and 3D alignments. Cross correlations are computed in the frequency domain, and then normalized in the spatial domain using pre-computed image integrals (Lewis, 1995). Both packages offer GPU implementations, which typically offer speedups (runtime comparisons are documented in the *rcc-xcorr* package) if the input images are large enough (bigger than about 1,000 pixels per side). Images are always preprocessed before the template matching using nxcorrs. Preprocessing parameters depend on the input image pixel size, but for 16 nm input the image preprocessing steps with parameters are: (1) contrast normalization using Contrast Limited Adaptive Histogram Equalization (CLAHE) with a clip limit of 30 and a tile size of 32 × 32, (2) conversion to 32 bit floating point, (3) whitening using a Laplacian of Gaussian (LOG) filter with sigma equal to 4, and (4) normalization by subtracting the mean and dividing by the standard deviation scaled by the square root of the number of image pixels, Zero-Normalized Cross Correlation (ZNCC). We found that all of these steps were useful in reducing the overall number of outlier cross correlations.

### 2D coordinate alignment

Multi-field-of-view (mFOV) tiles collected for the zebrafish retina dataset were 3,876 × 3,376 pixels (15.5 × 13.5 μm^2^ at 4 nm). Cross correlations between neighboring images were performed by using the entire destination image as the main image and a crop of the nearest quarter of the neighboring source image as the template image. Cross correlations for any tiles that had grayscale variance below 1 (very low contrast, for example fully zero images) had their associated deltas automatically assigned as outliers. For the first pass of the alignment tolerances were 768 × 768 nm^2^ for intra-mFOV comparisons and 15,440 × 13,440 nm^2^ for inter-mFOV comparisons, almost the size of a full tile because mFOVs could sometimes overlap by almost a full file. For the second pass, the tolerances around each median delta were 64 × 64 nm^2^ for intra-mFOV comparisons and 4,480 × 4,480 nm^2^ for inter-mFOV comparisons. A much larger inter-mFOV tolerance is necessary because errors in stage movement can in the worst cases accumulate to about 4–5 μm.

### 2D gradient and brightness correction

The two pass tile gradient and brightness correction relies on heuristics to determine outlier tiles. Because the overall brightness and contrast varies between sections, the heuristics must rely on the initial measure of the overall section histogram, and use that to determine a suitable range for considering tiles as inliers. Most outlier tiles are rejected simply by their mode relative to ranges determined from the overall section histogram. Additionally however, connected components are applied to each tile for all pixels above and below threshold values determined from the histogram, and some further tiles are rejected if they contain any large components (more than 20% of the image, for example) that are below this threshold. This is useful in rejecting tiles that might only partially overlap with the dark silicon wafer background, or tiles that contain many darkly stained membranes. Including either of these can cause artifacts in the final mean tiles. Mean tiles for the first pass are box filtered with a box size of 1 × 1 μm^2^. Larger sizes are not useful, because the gradient removal method is also useful for removing artifacts involved with the overlap area between tiles being image twice, and this overlap size is typically 1–2 μm. After the filtering, tiles are divided by their mode in order to create the gradient factor scale correction. For the second pass, the heuristics remain the same, but are now based on histograms computed from the gradient corrected tiles. Instead of filtering after the average tiles are created, the mode value of each tile is taken and then the overall image mode is subtracted from this, resulting in the tile brightness correction factors.

For the final sub-tile-based brightness correction, tiles are typically broken into 3 × 3 sub-tiles and histograms are computed and compared using ZNCC. Adjacency matrices created for the histogram shift linear system solver (LSS) are typically generated using the top 32 best matching histograms, resulting in a total number of neighbors that is 32 times the number of sub-tiles. For this reason, adjacency matrices could become prohibitively large for larger tissue sections (longer than about 200 μm per side), so for these sections sub-tiles were divided into randomized groups of 5,000 sub-tiles (without replacement) and the correction factors for the groups were solved independently. In order to prevent disconnected components in the adjacency matrix, minimum spanning trees of the graphs containing all pairwise comparisons and edge weights given by the corresponding correlation distances were computed. Edges from the minimum spanning trees were forcibly added, even if some of the edges were above the minimum correlation distance threshold for the histograms to be considered neighbors. The solved correction factors are single brightness offset correction factors for each sub-tile. Therefore, in order to avoid edge artifacts at the subtile boundaries, the solved brightness correction factors are fit with a 2D polynomial surface for each tile before being applied to the image tiles. This means the final correction factor is a smooth 2D polynomial surface for each tile. For the final section image montaging, feathering, or “ramps,” between tiles was used with a ramp distance of approximately 0.5 μm for ramps between tiles within the same mFOV and 3 μm for ramps between mFOVs.

### Tissue detection

The tissue in the EM sections was detected using a convolutional neuronal network closely following the UNet architecture (Ronneberger et al., 2015) with a single output class implemented in PyTorch (Falcon, 2019; Paszke et al., 2019). Our architecture differed from the original in three ways: (1) we used zero padding such that input and output of each convolution had the same image dimensions and thus removed the necessity of cropping in the skip connections; (2) dropout with factor 0.5 was introduced for the convolution prior to the last max pooling step and the convolution prior to the first upsampling operation to prevent overfitting (Srivastava et al., 2014); (3) we used the sigmoid non-linearity in the output convolution for probability outputs.

During training, the data was augmented with flip and transpose operations, intensity augmentations [i.e., multiplication with uniform random values in [0.8, 1.2), addition of uniform random values in [−0.2, 0.2) and Gaussian noise (zero mean, unit variance)]. Prior to the padding and cropping of the samples to the network input size of 256 × 256 pixels, each section was normalized to zero mean and unit variance. All augmentations and the normalization were implemented such that zero values were preserved.

The network was trained with 320 samples for 25,000 steps (each step with a batch size of one), the binary cross entropy loss function, the Adam optimizer with default parameters and a learning rate of 10^−6^ (Kingma and Ba, 2015). The validation dataset consisted of 80 samples. Ground truth samples were manually annotated by labeling tissue areas in full sections or sub-sections using webKnossos (Boergens et al., 2017). The labeling time depends of the ratio of tissue to background (e.g., bare Epon or silicon wafer) to be labeled and typically requires at most a few minutes per section. For inference, we used the best checkpoint with respect to the validation loss. The final tissue mask was generated by thresholding the inference output. We chose the threshold such that it minimized the binary cross entropy on a test set. Inference and training was performed with a downsampled pixel size of 1,024 × 1,024 nm^2^.

### 3D rough alignment

Although other feature detection algorithms are possible, the rough alignment and order solving utilized Scale Invariant Feature Transform (SIFT). Keypoints and descriptors in further downsampled images, typically 128 or 256 nm resolution, were detected using the opencv (Bradski, 2000) implementation with the default parameters. Features were matched by using the Lowe ratio test (Lowe, 2004), with the ratio of the second closest neighbor to the closest neighbor equal to 1.42, a conservative threshold. Matching keypoints were fit with constrained affine models using linear regression (least squares) with Random Sample Consensus (RANSAC) (Fischler and Bolles, 1981). During the initial order-solving, rigid affines were always utilized (see *Constrained Affine Transformations* below) with a RANSAC tolerance of 5.12 μm. For the zebrafish retina dataset, rigid affines with the same tolerance were also used for computing the rough alignment. For the zebrafish retina dataset, neighbors were considered as a bad match if the number of matching SIFT features, meaning they were considered matching by both the Lowe ratio test and by the affine RANSAC, fell below a threshold of 32 matches. In addition, heuristics were applied to prevent some spurious matches, which were maximum allowable translation for the affine fit and a minimal radial distribution of the keypoints. For the zebrafish retina only the maximum translation heuristic was utilized with a value of 196 μm.

For application of the rough alignment LSS, uniformly spaced (hexagonal layout) grid points were utilized with a spacing of 25 μm. The grid points for all neighbor comparisons (four neighbors in each direction of the section ordering) were evaluated with the fitted affine transforms, effectively converting the affine transforms into 2D vector fields. Any grid points that were more than 60 μm way from a matching SIFT point were discarded before application of the rough alignment LSS. Before application of the rough alignment LSS, all vector fields were converted to use the centroid positions of the grid, i.e., the Voronoi points. We found that solving the centroids instead of the actual grid locations resulted in more 2D smoothness of aligned images. For the zebrafish retina (35 nm section thickness), the rough alignment LSS was applied blockwise in z, using overlapping blocks of 721 sections (25 μm) of context and for each block of 241 sections (8 μm) being solved. For each block solution, the per-section and per-grid point resolved x,y deltas were fitted with a line (including offset) and this trend was removed from the deltas for each resolved grid point independently. This can be done for the rough alignment LSS because the adjacency matrix in this case is linear, i.e., the indices into the adjacency matrix increased were the exact relative z-coordinates of the sections being solved.

### Constrained affine transformations

The *msemalign* package allows fitting affine transformations at various levels of constraint. Fitting full affine transformations in 2D results in 6 total free parameters (including translation) and is done easily by applying a linear regression (least squares). Analytical solutions that find affine fits with constraints are particular instances of the more general Procrustes analysis. Specifically, affine transformations can be decomposed into the 4 sequential operations of rotation (angle), scaling (x and y), shearing (x and y) and translation (x and y). Translation is easily solved as the difference between source and destination point means after the 2 × 2 (unaugmented) affine matrix is computed. If both scale and shear are constrained to be zero, this is known as the orthonormal Procrustes problem, or rigid-body alignment. An optimal solution is given by the Kabsch–Umeyama algorithm, implemented in the *msemalign* package. This algorithm can also allow for a scale parameter, but it must be the same scale in x and y. Introducing an intermediate level of constraint, specifically that allows different scales in x and y, but does not allow for shear, is a more complicated analytical problem known as the orthogonal but not orthonormal Procrustes problem (Everson, 1997). Although analytical solutions do not exist in higher dimensions, the 2D problem can be expanded out from the involved matrices and solved analytically. This provides an optimal solution that specifically allows for different scale factors in x and y, but does not fit any shear. For tissue sectioning it is physically realistic to require different scale factors in x and y, because compression of the sections occurs in the direction of cutting rather than in the orthogonal direction, and the amount of compression can vary between sections.

### 3D fine alignment

For the zebrafish retina dataset, grid points chosen for computing nxcorrs were again uniformly (hexagonal arrangement) spaced with a spacing of 16 μm. For alignments with a larger xy area per section, we found it better to use a larger spacing, 24 or 32 μm, mostly because we prefer the initial fine alignment to be relatively rigid (enforced by the affine filtering of the vector fields) up to the scale of about 100 μm. We found that non-linear deformations on average only became substantial at distances beyond 100 μm, meaning that most physical deformations that result from the sectioning are still locally rigid. At each grid location neighboring slices were cropped centered on the grid locations to run nxcorr-based template matching. For the zebrafish retina, source (image) crops were 32 μm and destination (template) crops were 4.8 μm. Four sections in each direction of the section ordering were used as neighbors for each section. To help reduce outlier deltas and account for any tissue where there happen to be more local non-linearities in the deformations, for example torqueing of the tissue, template matching is done by rotating the templates through a range, for the zebrafish retina dataset with [−15, 15] in increments of 3 degrees. For sections that have artifacts such as tears or large folds, or bad image deformations that are larger than about 50 μm, the *msemalign* would try to correct these first and in isolation, before any 3D alignment is attempted. With good artifact correction, this simplifies the 3D alignment, but still allows inclusion of sections that would otherwise need to be excluded.

Outlier detection in the 2D vector fields for the fine alignment relies on a series of heuristic methods. The initial test is simply to apply RANSAC to a model of an affine transformation containing quadratic terms and use the RANSAC classification of outliers. Typically a looser RANSAC tolerance is utilized, for the zebrafish retina dataset 12 μm. The final step is to re-consider some outliers that maybe have been rejected by this model by comparing each delta against the angle and magnitude deviation of the most similar vectors in the neighborhood. For the zebrafish retina dataset, the diameter of this neighborhood was 57 μm. Any grid points that were flagged as outliers are then replaced with estimates based on a model of rigid image deformation, Moving Least Squares (MLS) (Schaefer et al., 2006), which was easily adopted for interpolation / extrapolation instead of deformation. The replaced outlier deltas are weighted by 0.5 relative to the inlier deltas when applying the delta LSS.

For initial fine alignment we applied an affine filter (discussed in results) with a box size of 61 μm. The delta LSS for the fine alignment essentially works the same as for the rough alignment, except that we found it useful to weight the input deltas depending on the neighbor distance. The relative weights used for the n ± 1 up to n ± 4 comparisons were 1.0, 1.0, 0.4, and 0.1, respectively.

Many parameters are adjusted for the ultrafine alignment. For the zebrafish retina ultrafine alignment, a much finer grid spacing of 2.0 μm was used. Two neighbors were used for nxcorr-based template matching in each direction of the solved ordering. Source (image) and destination (template) crop sizes were 12.8 and 3.2 μm, respectively. The affine filtering was disabled for the ultrafine alignment, but instead ridge regression was utilized with the delta LSS with an L2 regularization factor of 0.05.

### Export

Both the fine and ultrafine exports are implemented blockwise using an inverse coordinate transform method as described in Results. This allows for scalability of the exports, particularly for the final export at 4 nm. For square-mm-sized sections, the rough bounding box was typically divided into 4 × 4 blocks for 16 nm exports and 16 × 16 for the final 4 nm export. To avoid edge artifacts, the blocks required a very small amount of overlap, usually set to 8 pixels in x,y. For the fine and ultrafine warpings, coordinates corresponding to every pixel must be warped, meaning that the 2D vector fields that describe the warping must be interpolated to be pixel-dense. Although a more complicated interpolation algorithm (for example MLS) could be utilized, we found that simple bilinear interpolation was sufficient for interpolating between the vector field grid points and was also much less computationally costly. The exports were cropped to the bounding box of the grid points to avoid extrapolation. So that no tissue areas were cropped from any sections, grids points were always spaced to fully cover the tissue.

Blockwise exporting for 2D montaged sections is also supported. This case is relatively simple, as it can simply montage tiles that have coordinates within the block, again utilizing some overlap to avoid edge effects related to the tile feathering (for the zebrafish retina dataset 8 μm in x,y).

## Results

The *msemalign* pipeline manipulates images downsampled from the pixel size in which they were acquired. Most typically, images are downsampled from the acquired pixel size of 4 nm to 16 nm. In order to economize on compute time, further downsampling may be possible without loss of alignment quality, depending on the tissue type. The transformations computed at 16 nm are scaled and then applied to the 4 nm data to create the final aligned dataset export. Typically we section embedded tissue blocks at a nominal section thickness of 35 nm. Sections are collected onto silicon wafers using the GAUSS-EM methodology (Fulton et al., 2023), wherein the ordering of the sections from the tissue block is not preserved, and the sections are randomly oriented on wafers. The overall orientation angles of the sections are typically uniformly random. Regions of interest (ROIs) to be imaged are positioned over each section on a light microscope overview image of all sections on a wafer. The acquisition results in a collection of small image tiles (15.5 × 13.5 μm^2^, 4 nm pixel size, 8-bit grayscale) for each section, acquisition-based x,y image coordinates for each tile and coordinates of the ROI within the image coordinate space of each section. The total number of image tiles for an entire petabyte-scale dataset is on the order of 100 million.

**Figure 1:**
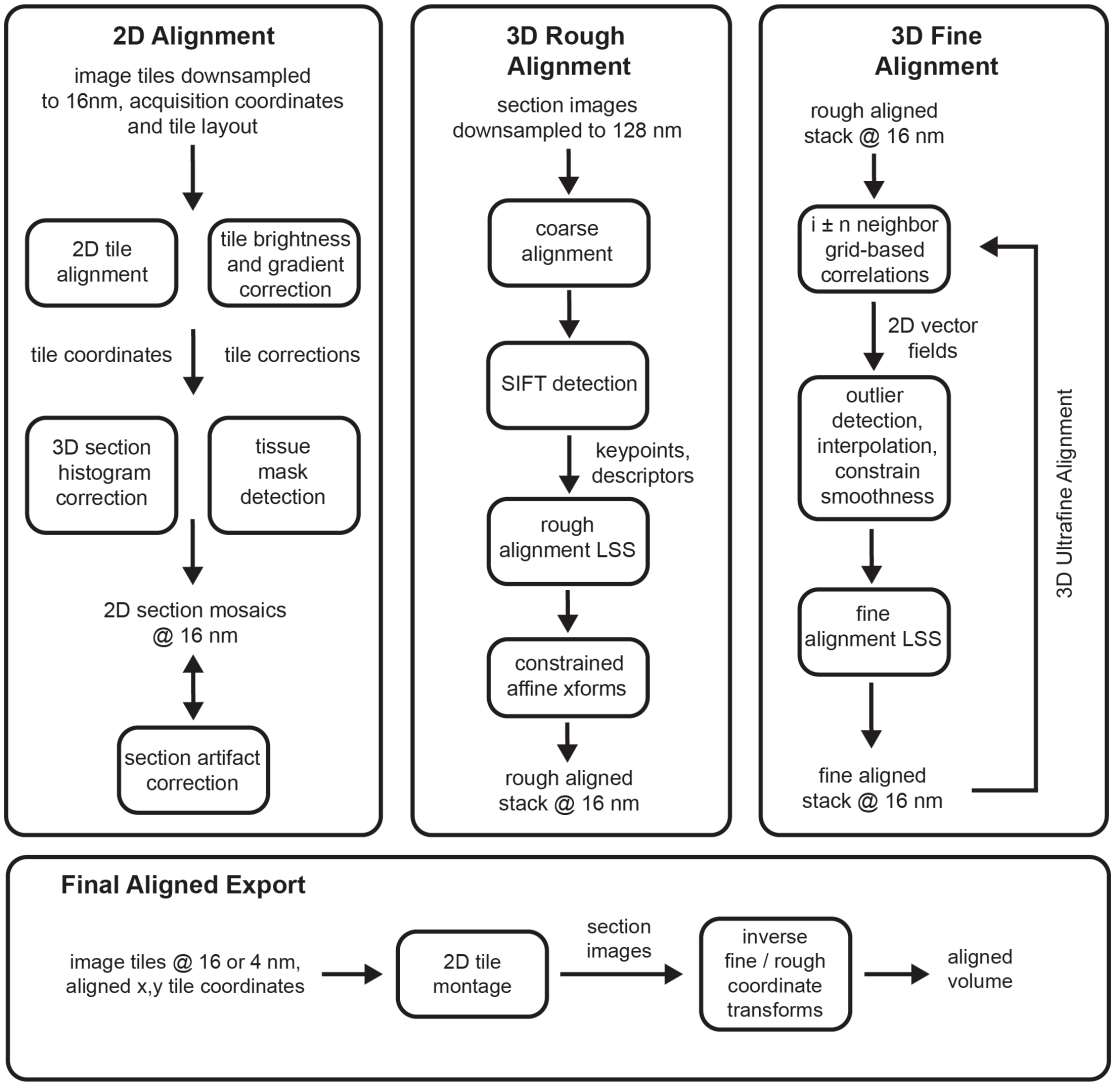
Flowcharts showing an overview of the major steps involved in the entire *msemalign* pipeline.

The *msemalign* pipeline (Figure 1) can be divided into four major components: (1) 2D section alignment: creates single large mosaics of individual sections by aligning and balancing small image tiles, (2) 3D rough alignment: uses image features to recover the ordering of the sections [if applicable (Fulton et al., 2023)] and then roughly align the 2D section images using a single affine transformation per section, (3) 3D fine alignment and ultrafine alignment: iteratively refine the rough alignment by computing template-based correspondences between neighbors at fixed grid locations, and (4) export: applies inverses of the computed transformations block-wise on output grid coordinates to create the final elastic transformation that warps each section into 3D alignment. The following sections discuss each of these major components in detail.

### 2D alignment

**Figure 2:**
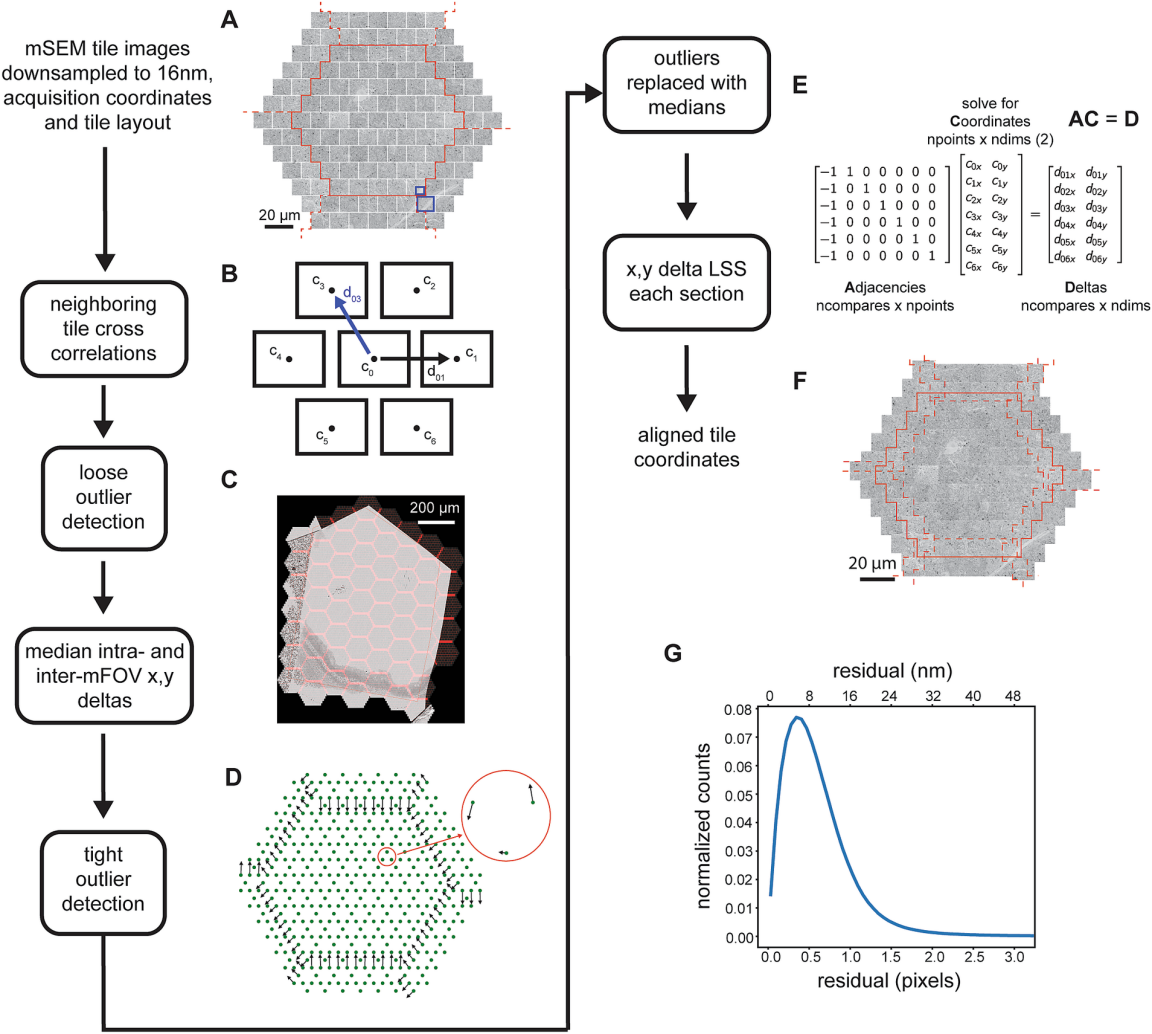
Detailed flowchart of the steps involved in the 2D image tile registration. **(A)** All image tiles for a single mFOV (in this case with 61 tiles, outlined in solid red) and with tiles for two of the closest rings included from the 6 neighboring mFOVS (dashed red lines). **(B)** Schematic showing the neighbors for a single tile and example of the x,y deltas. The blue line corresponds to deltas as calculated by comparing the blue highlighted areas in **(A)**. **(C)** mFOVs for an entire section with the overlapping areas between tiles and between mFOVs highlighted in red. **(D)** Example of median deltas calculated across all mFOVs for a single section. Red circle shows a zoomed inset of the intra-mFOV median deltas. **(E)** Matrices illustrating the x,y delta LSS representing just the tiles depicted schematically in **(B)**. **(F)** Example of the tiles shown in **(A)** after they have been positioned based on the solved coordinates. **(G)** Distribution of residuals from the x,y delta LSS.

The 2D alignment procedure is tied to the acquisition format of the data acquired by the Zeiss mSEM microscope (Eberle et al., 2015), containing 91 electron beams arranged as a hexagon that simultaneously image tissue sections. After an area encompassing the 91 beams, known as a multi-field-of-view (mFOV), is imaged, the sample stage is moved in order to acquire images for the next adjacent mFOV. mFOVs are then tiled to acquire images covering the previously defined ROI (Figure 2C). The tiles within each mFOV are overlapped with one another by about 1–2 μm based on beam coordinates, and between mFOVs by about 10 μm based on stage coordinates. The process is repeated until image tiles that cover the ROIs of all serial sections on a wafer are acquired. The *msemalign* 2D alignment assumes the mFOV structure and associated hexagonal layout of the tile images, although it could easily be adapted for alternative tile layout patterns. Most of the 2D alignment is concerned with finding optimal translations and brightness gradient corrections of the individual beam tile images so that the final section images appears as smooth single large image mosaics in which tile boundaries are no longer visible.

The x,y coordinates of individual tiles and the hexagonal layout of the tiles within and between mFOVs is used to determine the nearest neighbors for each tile. The process is parallelized by loading tiles for a single mFOV, but including perimeter tiles from neighboring mFOVs (Figure 2A). With the exception of tiles around the edges of an mFOV, the hexagonal layout dictates that each tile has 6 immediate neighbors. Typically a full tile is compared against the closest quarter crop of the neighboring tile (Figure 2A, blue boxes) using normalized cross correlation (nxcorr). The peak matching location of the nxcorr is used to find optimal x,y deltas between the image tile centers (Figure 2B). Using normalized cross correlations as a template matching method usually produces the optimal matching location, but still generates some outliers that are not. Typically this occurs for one of two reasons: (1) the tiles being compared contain very little structure, for example in areas of the serial sections that either do not contain tissue or are in the center of cell bodies or blood vessels; (2) topographies in the resulting cross correlation image that are not peak-like, for example ridges or plateaus (Scheffer et al., 2013; Buniatyan et al., 2017). Ultimately some type of optimizing solver must be applied to the x,y deltas obtained between neighboring tiles in order to compute optimal tile positions for all tiles in each section. For our purposes we have chosen a linear solver that we call the linear system solver (LSS), which is used at multiple steps throughout the pipeline. This solver is only robust to small amounts of noise and thus can be overwhelmed by any significant percentage of outlier cross correlation x,y deltas. For this reason, outlier x,y deltas must be detected and replaced with a reasonable estimate delta before application of the 2D x,y delta LSS.

Outlier x,y deltas from the neighboring 2D cross correlations are detected using a two-pass procedure. In the first pass, deltas are compared against those expected based on overlap parameters from the acquisition, either for tiles within the same mFOV (intra-mFOV) or between mFOVs (inter-mFOV). Because the inter-mFOV comparisons are subject to positioning errors in stage movements, which are much larger than intra-mFOV errors in the 91 beam positions, the tolerance for the inter-mFOV comparisons must be greater. Still, during the first pass relatively large tolerances are used, primarily because stage-movement associated errors are not known *a priori*. Thus, the first pass only removes egregious outliers. Deltas from the first pass are used to compute median x,y deltas for each adjacent tile (Figure 2D) using a series of mFOVs that were acquired sequentially in time (but can be from different sections). The median deltas then serve two purposes: first as the values around which to apply a tighter tolerance for rejection of outliers and second as replacement x,y deltas for all detected outliers. This second pass then removes additional outliers and replaces outliers detected in both the first and second pass with a reasonable median replacement value.

After detection and replacement of outliers, all x,y deltas for a given section are input into the 2D x,y delta LSS (Figure 2E). The LSS without regularization simply finds a linear regression solution (a least squares solution) for an over-specified system. The system consists of a matrix describing the neighbors, or adjacencies, which contains exactly two nonzero values in each row, one and minus one. This effectively creates a difference between neighboring tiles in each row. This matrix is multiplied by the unknown “global” x,y coordinates, the differences of which are the previously measured x,y deltas. A weighting matrix can be applied so that, in the case of the 2D alignment, outlier deltas that have been replaced with median values are given a lesser weighting. Although over-specified, the system will typically not have a unique solution, and solutions can contain offset, linear trend, or low frequency oscillation biases. For the case of the adjacency matrixes that result from neighboring comparisons in a hexagonal 2D tessellation created by the mSEM tiles, the only bias we observed was an offset. This can simply be removed by subtracting the mean value of the solution, resulting in global coordinates that are used to montage the tile images into a large image mosaic of the entire section (Figure 2F, aligned mosaic of a single mFOV), with the x,y coordinate origin near the center of the mosaic image. The residuals remaining between the solved x,y deltas and those input to the 2D x,y delta LSS provide an indication of the overall quality and continuity of the mosaic. The mean value of the overall residual distribution for the 2D alignment of the zebrafish retina dataset (Figure 2G) was 1 pixel (16 nm) and 99% of the residuals were below 5.9 pixels (95 nm).

### 2D gradient and brightness correction

**Figure 3:**
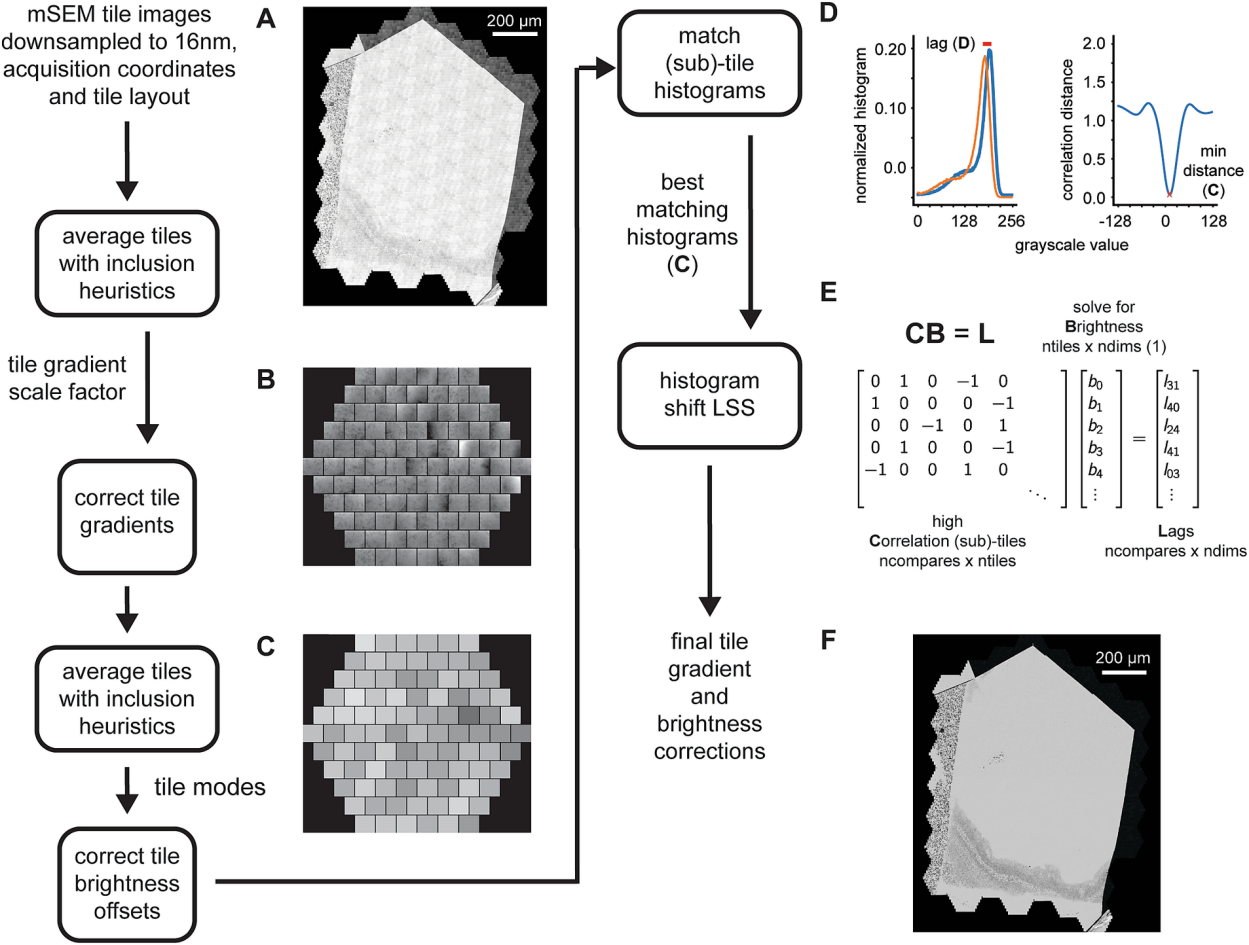
Detailed flowchart of the steps involved in the 2D tile gradient and brightness correction. **(A)** Example section image showing the pattern of tile gradient and brightness mismatches that repeats across the mFOVs. **(B)** Output of the first pass mFOV averaging method illustrating the gradients measured for each tile relative to their mode. **(C)** Output of the second pass mFOV averaging method depicting the relative brightness measure for each tile. **(D)** Examples of two normalized histograms from sub-tiles used for the final refinement of the gradient and brightness correction. The red line shows the lag (left) that corresponds to the minimum correlation distance for the normalized cross correlation between the histograms (right). **(E)** Exampled matrices for the linear system solved by the histogram shift LSS in order to calculate the fine sub-tile brightness corrections. **(F)** Same section image as in **(A)** but after all the tile gradient and brightness corrections have been applied.

The mSEM acquisition is subject to artifacts that stem from inhomogeneous areas in the electron collection scintillator and from electron optical inhomogeneities between the 91 electron beams. This results in visible artifacts in the montaged section images. Tiles within each mFOV can contain brightness gradients within single tiles and brightness offsets between tiles. The net result is a section image that has a clear repeating pattern (that is consistent between mFOVs within a section) which reflects these gradients and brightness differences (Figure 3A). In the worst cases it may be difficult to observe the tissue itself in the overview of each section image because the image is dominated by this pattern of artifacts.

The gradient and brightness correction is performed using a two pass method, the first pass corrects for intra-tile gradients and the second pass for inter-tile brightness offsets. The procedure does not require solved tile positions, therefore the correction steps can be run in parallel with the alignment steps (Figure 2). In the first pass, tiles corresponding to the same intra-mFOV positions are averaged together, resulting in an “average 91-tile mFOV” for each section. The inclusion of tiles into the average is subject to exclusion heuristics that will reject, for example, any tiles that are mostly saturated near the ends of the overall section grayscale histogram. We noted that the gradients are mostly linear within the middle of the grayscale range, meaning that averaging mFOV tiles results in a good estimate of the gradients for each tile, as long as each section has a sufficient number of mFOVs. The estimates are only consistent when averaging tiles within the same section because, for each section, a beam alignment to the detector and autofocusing/autostigmation are performed that can change the gradient pattern within each mFOV. The average tiles are then blurred and divided by their respective modes, creating scale factor images for each tile (Figure 3B). The corresponding tiles in each mFOV are divided by the scale factors to correct for the gradients. The tiles corrected by the first pass are then averaged again using the same procedure. This time, however, the mode for each tile is calculated to correct for brightness differences between tiles. The difference around the mean mode is used as a brightness offset correction factor (Figure 3C, single integer grayscale value per tile) that is used to correct the remaining brightness offsets between tiles.

We observed that some differences at tile boundaries may still remain due to slight differences in the grayscale histograms of neighboring tiles, so we employ a third procedure on top of the two pass correction method. This procedure computes histograms for sub-tiles within each tile. All possible pairwise histograms are then compared to one another using normalized cross correlation between the histograms (not the images). This results in a large set of cross correlation distances (where 0 indicates identical histograms) and their associated lags (Figure 3D). The cross correlation distances are rank ordered, and then the top-n matches are selected and considered as “neighbors” in the adjacency matrix (Figure 3E, high correlation sub-tiles) of the histogram shift LSS. These adjacencies multiplied by the unknown optimal sub-tile brightness values equal the corresponding computed lags. In order to prevent the possibility of unconnected components in the adjacency matrix, a minimum spanning tree is calculated over the entire graph of correlation distances, and any corresponding edges required to connect the minimum spanning tree are added to the adjacency matrix. With this addition, we observed that the histogram shift LSS only demonstrates an offset bias. This bias is removed by subtracting the overall mean from the solved brightness values, resulting in per-subtile integer brightness offsets. These offsets are then removed from the corresponding sub-tiles.

Finally, the 2D section images are montaged, using the solved coordinates and after applying all gradient and brightness correction factors. A feathering technique is used to blend the tile borders together. The feathering is computed by applying 2D ramps at all of the overlap locations, with the length of the ramp set as a parameter, and different depending on if the feathering is being applied to tiles within the same mFOV or between different mFOVs. The end result of the 2D alignment portion of the *msemalign* pipeline is a stack of 2D aligned and brightness/gradient corrected section images (Figure 3F). Primarily due to the difficulty of inverting the feathering procedure when montaging, in the current *msemalign* implementation this image stack is exported and stored on disk at a pixel size of 16 nm (~63 TBytes for a 1 PByte dataset acquired at 4 nm).

### 3D contrast and brightness correction

Our current acquisition automation strategy does not optimize EM acquisition parameters such as dwell time for each individual section (although sections can be re-imaged if needed post-hoc). This means that small variations in section thickness that can occur when cutting ultrathin sections at a nominal thickness of 35 nm results in variability of the dynamic range and therefore variability in the contrast of the section images. This necessitates a further correction, balancing the overall brightness and contrast among the section images. We found that contrast balancing methods such as matching to a uniform grayscale distribution or automatically scaling each image to the full range did not work well, in part because each image section can contain areas of image data that is not from the tissue of interest. These areas include resin-only areas, areas of iron particles (Fulton et al., 2023), debris or charging artifacts and locations entirely outside of the sections (e.g., the silicon wafer substrate). This can skew the result of histogram matching procedures such that the matching is not optimal within the tissue containing regions of each section. We found that it is best to disregard non-tissue containing areas, which therefore necessitates a method of masking out these areas of the image.

**Figure 4:**
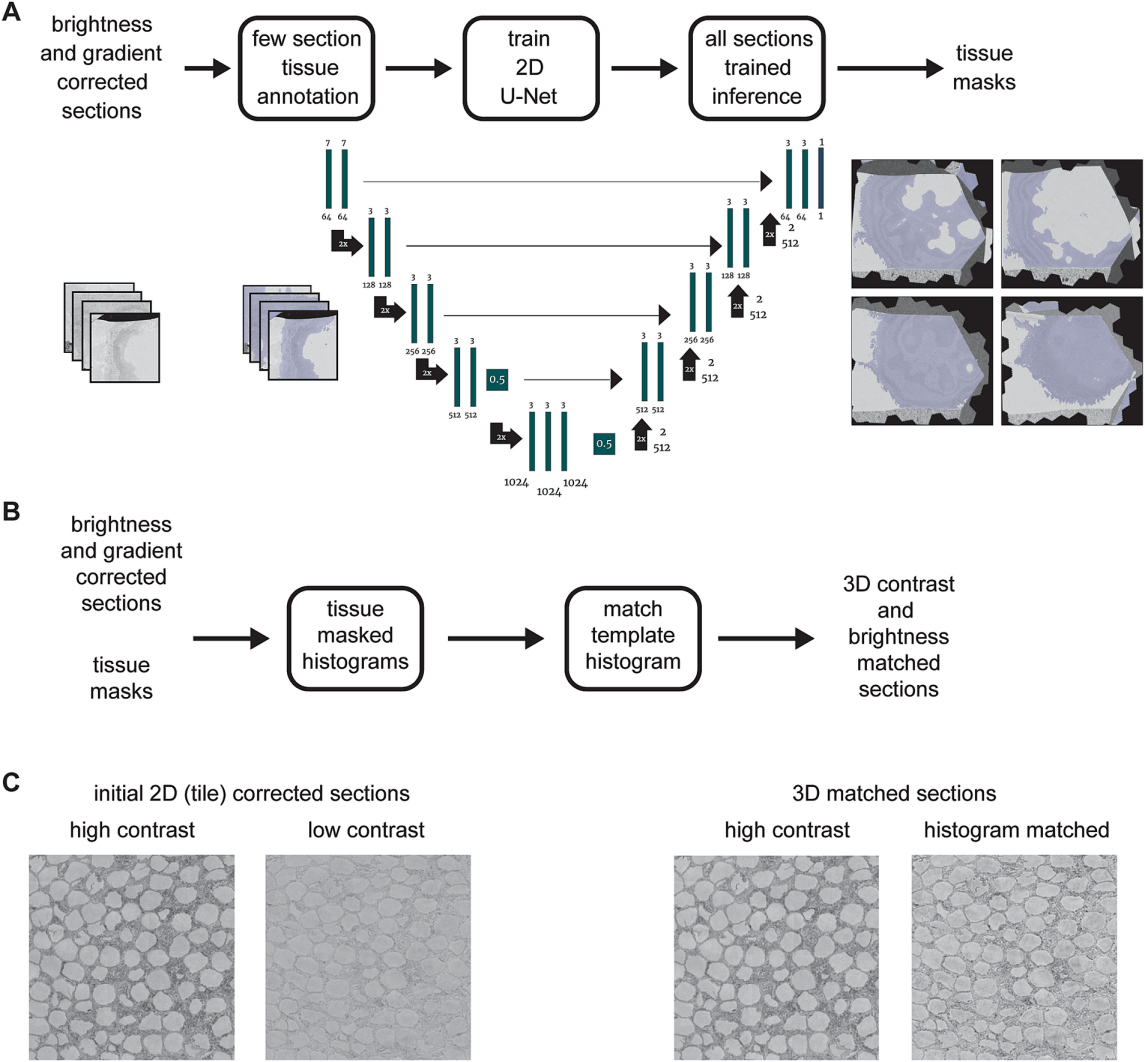
**(A)** Flowchart and schematic of the tissue detection portion of the *msemalign* pipeline showing the 2D U-Net architecture. Training tissue masks are manually labeled (left blue overlays), but the majority of the tissue masks are generated (right blue overlays) by an inference pass of the trained U-Net. **(B)** Flowchart of the 3D section image brightness and contrast correction, accomplished by using histogram matching to a manually selected high contrast section template histogram. **(C)** Samples before and after correction showing corresponding changes to a high contrast and a lower contrast section.

In order to avoid manual labeling the tissue areas within each section image, we utilized a simple machine learning method to generate masks of tissues areas for all sections (Figure 4A). The procedure involves manually labeling tissue areas in a small subset of sections as training data for a 2D U-Net (Ronneberger et al., 2015). All sections are then run through an inference pass of the trained U-Net to automatically generate tissue masks for the entire dataset. Once the masks are generated, they are applied to the histogram measurement for each section, so that only pixels from within tissue areas are included (Figure 4B). Finally, a selected section that already had high contrast when it was acquired is used as a template histogram, and a histogram matching algorithm (Bourke, 2011) is then applied to all sections. This results in essentially no change for sections that already had high contrast, but a substantial improvement and consistency with other sections for sections that originally had lower contrast when they were imaged (Figure 4C).

### 3D coarse/rough alignment and order solving

Once large 2D section images have been created for the entire dataset, typically organized in the same way they were acquired, i.e., one section set per silicon wafer, the 3D alignment can proceed. In contrast to some previous alignment procedures (Saalfeld et al., 2012), we did not find it useful to consider both 2D and 3D alignment simultaneously, but instead rely on good 2D section montages to further constrain the 3D alignment so that it is only concerned with inter-section image warping, and not intra-section translation of individual image tiles. Before the acquisition, sections are typically collected onto several silicon wafers, each containing several thousand sections. At this point, if the GAUSS-EM acquisition procedure was utilized, the section ordering from within the original tissue block must be recovered for each wafer (Fulton et al., 2023). Alternatively, if the section ordering is already known, the remainder of the *msemalign* pipeline is no longer dependent to the particular serial sectioning or the SEM acquisition methodologies used, but can instead operate directly on a stack of 2D serial section images.

**Figure 5:**
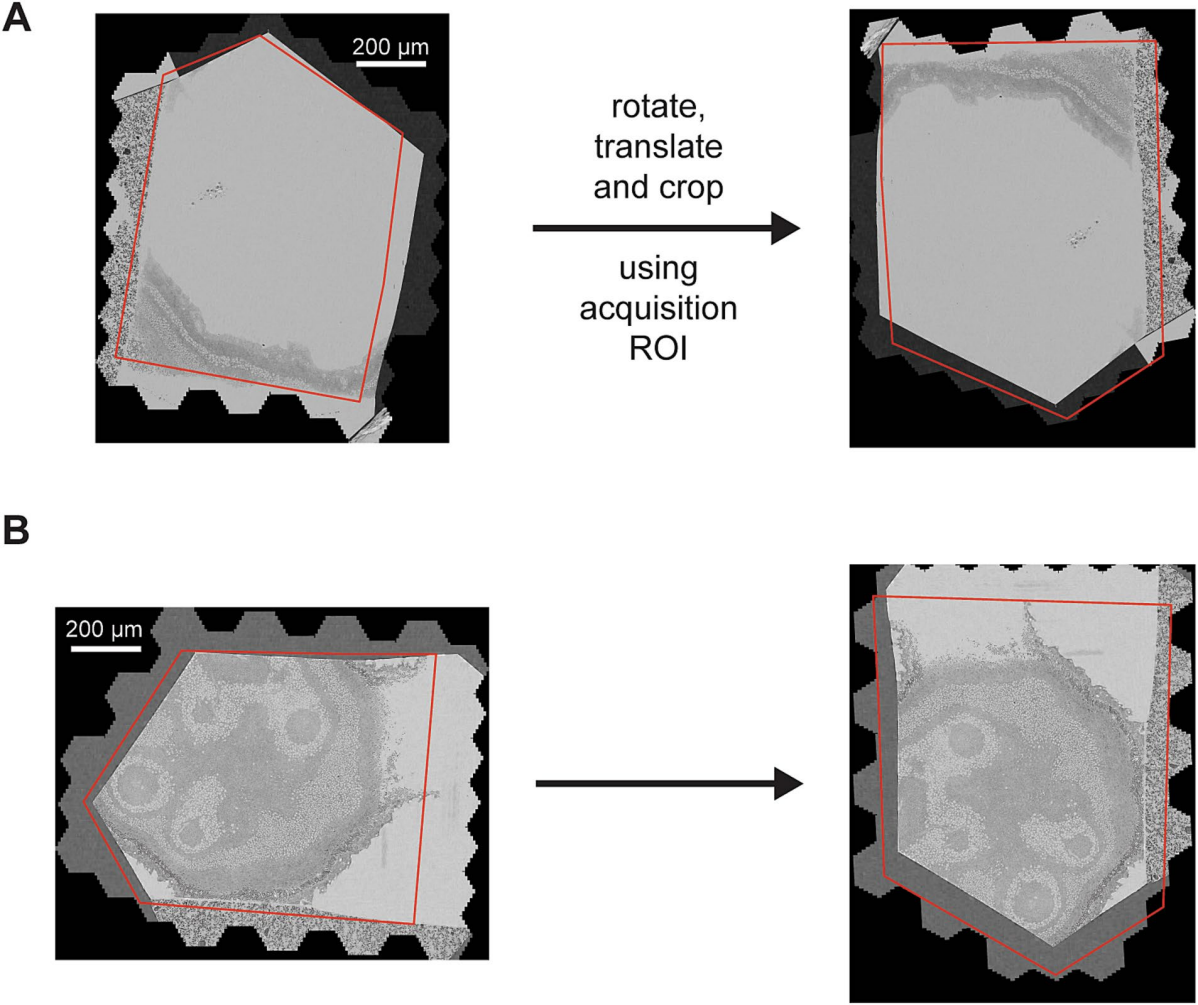
**(A,B)** Two different sections overlaid with the acquisition ROI (left) and how they appear after the coarse alignment (right) that rotates the sections by the angle of the ROI and translates the center of the ROI to the center of the cropped image (the rough bounding box).

The rough alignment begins by re-orienting all the sections by the angle and center determined by the acquisition ROIs, termed coarse alignment (Figure 5). Because the coarse alignment is based on ROIs that were placed using a light microscope overview (0.913 μm pixel size) of each wafer, they are subject to positioning and calibration errors with respect to the EM-pixel space. The coarse alignment provides a reasonable starting point for the 3D alignment, but the variability associated with the ROI placement and calibration can still result in offset errors as large as 100 μm translation and/or 15 degrees rotation. Therefore, a further heavily constrained rough alignment is performed that computes single global affine transformations for each section, termed the rough alignment. After the coarse alignment is applied, all images are cropped to the same bounding box, the rough bounding box. The size of the rough bounding box is chosen conservatively so that no tissue areas of any section are cropped out.

### 3D rough alignment

**Figure 6:**
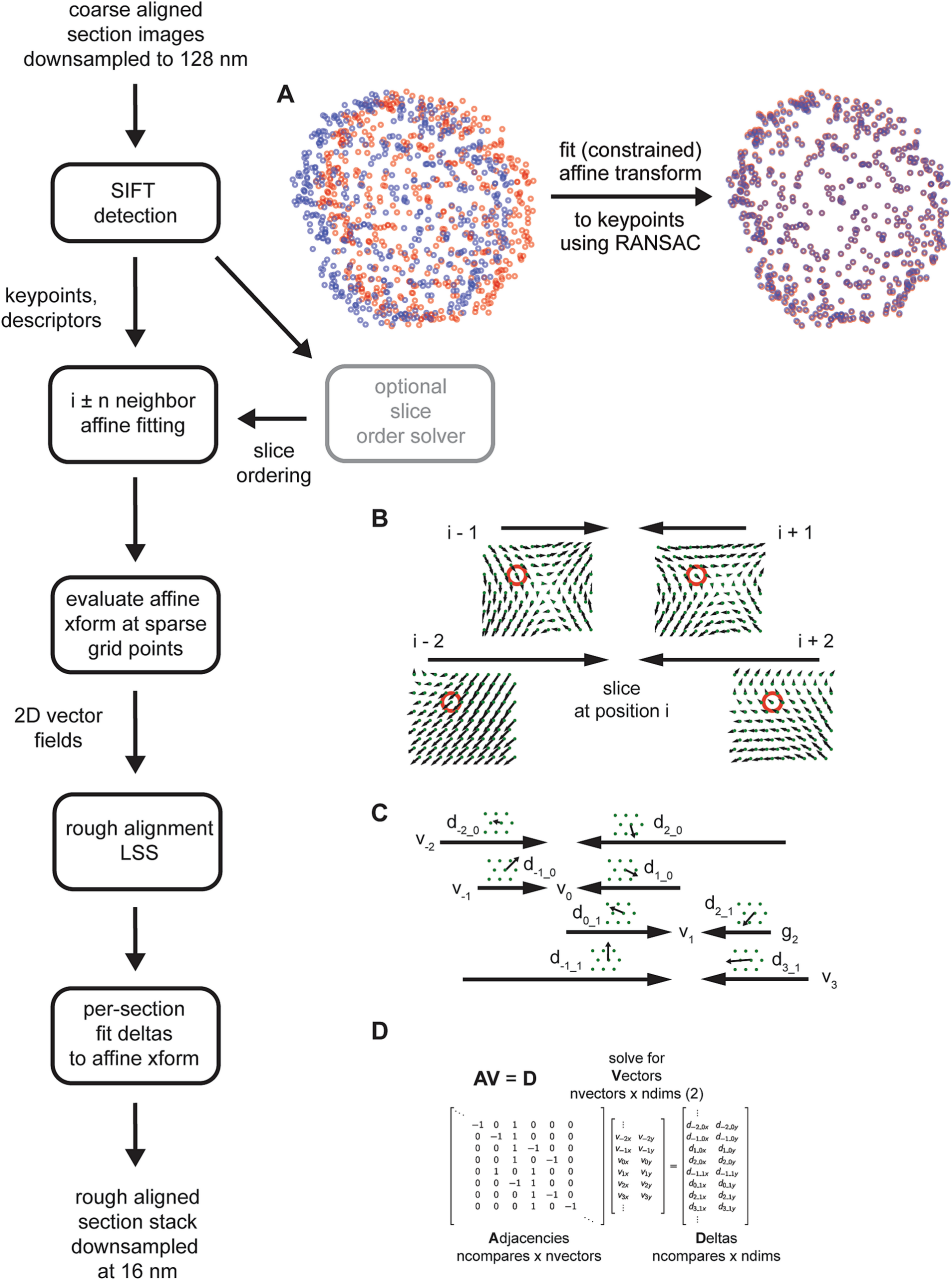
Detailed flowchart of the rough alignment, excluding steps required for solving the section ordering (light gray). **(A)** SIFT keypoints for two example neighboring sections in the zebrafish retina in their original locations based on the coarse alignment (left, blue and red points) and then after an affine transform is calculated and applied to the source keypoints (right). **(B)** Schematic demonstrating how the vector fields generated from the fitted affine transformations are solved independently per grid point (red circles depict a single grid point being solved) by applying the rough alignment delta LSS. Only the vector fields computed for a single section with two neighbors in each direction are shown. **(C)** Schematic similar to **(B)** but showing deltas computed from two different sections. **(D)** Matrices containing variables from the schematic in **(C)** that are solved by the rough alignment delta LSS.

Coarsely aligned 2D sections are downsampled further, and image features are computed on the downsampled images. Although several alternatives are available, the *msemalign* pipeline currently only utilizes Scale Invariant Feature Transform (SIFT) features. SIFT features comprise keypoints, pixel locations of salient features in the images, and descriptors, a 128-bit vector that encodes local information around the keypoint, in particular in a manner that is robust to local affine transformations (Lowe, 2004). SIFT descriptors from different images are matched to each other by computing the nearest neighbors between the descriptor spaces of the two images and then applying a threshold to the ratio of the distances to the nearest neighbor and to the second nearest neighbor, the Lowe ratio test (Lowe, 2004). The mapping of matching SIFT descriptors is not necessarily one-to-one, meaning that a destination image descriptor could be the nearest neighbor to multiple source image descriptors. Spurious matches, i.e., matches that do not really correspond to matching locations between images, are also possible. Therefore after potential matches are identified with the Lowe ratio test, the matching keypoints are fit with an (optionally constrained) affine model using the Random Sample Consensus (RANSAC) algorithm (Fischler and Bolles, 1981). The RANSAC algorithm removes outliers that do not fit within a parameterized tolerance to the affine model. Applying the affine transformation to the source keypoints from one section image maps them closely to the matching keypoints in a destination image that is a direct neighbor in the section ordering (Figure 6A).

The SIFT matching and RANSAC affine procedure is performed for the nearby neighbors of each section in the section stack, i.e., i ± n neighbors where n is a free parameter. Once all of these affine transformations have been calculated, they are evaluated on a fixed grid of points spanning the rough alignment bounding box (Figure 6B), creating 2D vector fields for each section comparison. A LSS is applied to the x,y deltas in the vector fields created by evaluating the affine transformations, but applied independently per grid point. In the case of the rough alignment delta LSS, the adjacency matrix represents neighbor comparisons for the entire z-stack for a single grid point (Figure 6C). The adjacency matrix multiplied by the “vector” coordinates for the unknown grid point are equal to the deltas measured at that grid point (Figure 6D). We found that the rough alignment delta LSS is susceptible to all forms of the potential biases (see *Constrained Affine Transformations* below). The solution to the rough alignment delta LSS essential reconciles all the neighbor comparisons to create a single warping vector field for each section. The *msemalign* strategy is to constrain warping at each step as much as possible, in order to gradually bring sections into alignment and thereby constrain the overall magnitude of the final warping that needs to be applied to each section. Consistent with this theme, instead of applying a warping based on the computed vector fields, the vector fields are refit with affine transformations. This produces a single global affine transformation for each section, the rough alignment.

### Constrained affine transformations

**Figure 7:**
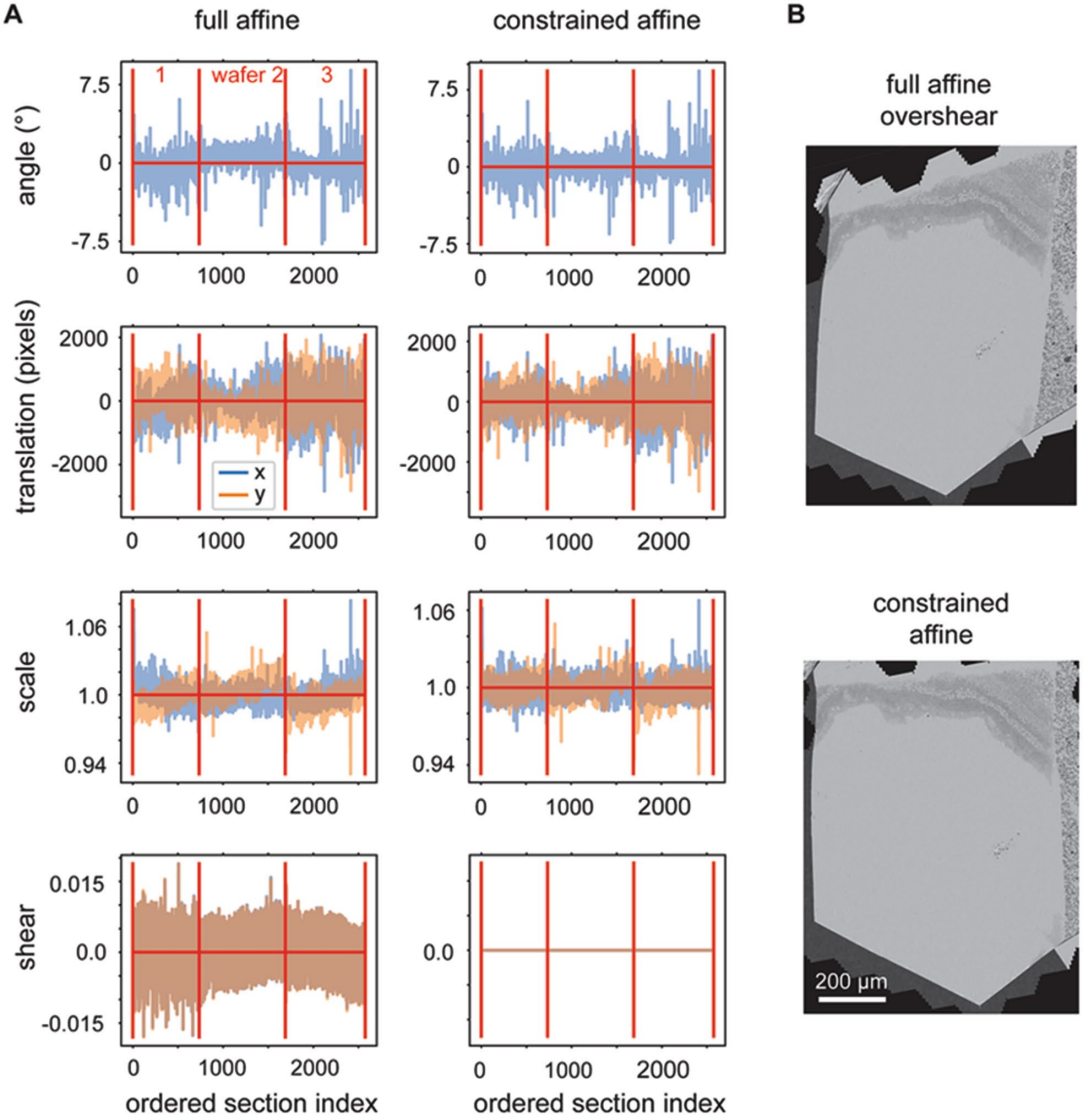
**(A)** Plots showing the variables decomposed from the solved rough affine transformations: angle, translation, scale and shear. The left plots show the decomposed values when full affine transformations were fit by utilizing all z-location deltas simultaneously. The right plots show the decomposed values when using a constrained affine that does not fit shear and when the rough alignment delta LSS is applied using overlapping blocks in z. **(B)** A section after rough alignment was applied that contains excess shear (top) and one after the rough alignment where the shear has been constrained to equal zero (bottom).

We observed that the rough alignment delta LSS is susceptible to offset, linear trend and low frequency oscillation biases. Likely the system is less constrained in comparison to the delta LSS utilized for the 2D alignment, because the graph that describes the neighbors (adjacencies) has a one dimensional (along Z) instead of planar (across X,Y) topology. This is because the rough alignment delta LSS is applied to the grid points independently, so nothing about the 2D position of the grid points is encoded into the adjacency matrix. In order to overcome the biases, linear trends (including offsets) are also fit and removed from the solution to each grid point before refitting the vector fields for each section with an affine transformation. When the affine transformations are decomposed into rotation, scale, shear and translation elements, it is evident that the procedure produces reasonable affine transformations (Figure 7A, full affine). On careful examination, however, two further issues become evident: (1) a low frequency oscillation, for the depicted dataset this occurs mostly in the scale fits, and (2) excessive shear components of the affine transform. We addressed the first issue by modifying the application of the rough alignment delta LSS so that it resolved deltas in overlapping blocks in z for each grid point, instead of all sections simultaneously. For the second issue, some sections become heavily sheared when fit with the full affine transformation, which reflects an unrealistic global deformation for our sectioning methodology (Figure 7B). We addressed the shear overfitting issue by finding the optimal solution for 2D affine transformations that constrain the shear to be exactly zero. An analytical solution of this application of the orthogonal, but non-orthonormal Procrustes problem (Everson, 1997) can be derived for 2D transformations. The decomposition of this more heavily constrained rough alignment procedure result in reduction of the low frequency oscillations and indeed resulted in exactly zero shear in the affine decompositions (Figure 7A, constrained affine).

### 3D fine alignment

**Figure 8:**
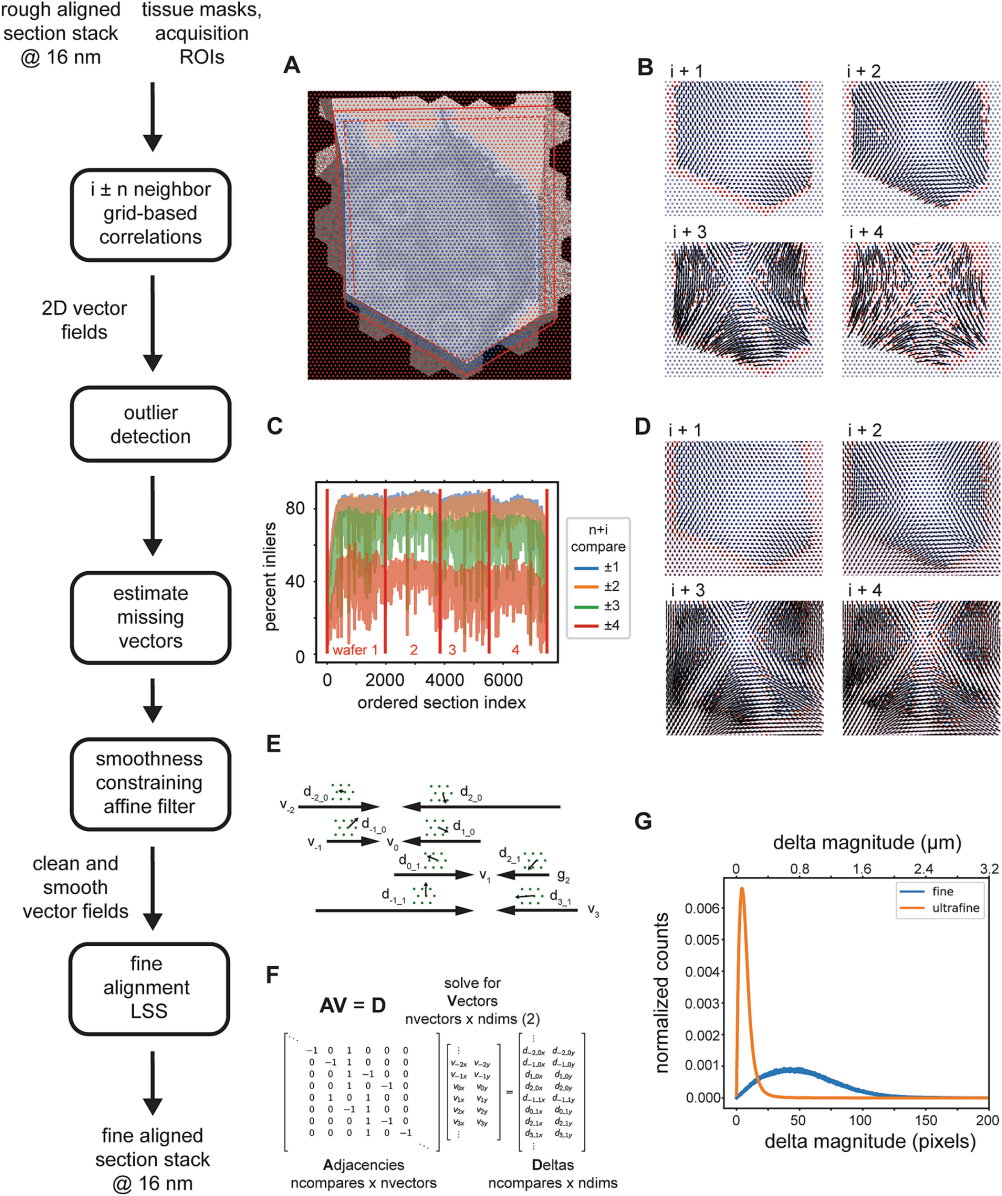
Detailed flowchart of the fine (and ultrafine) alignment. **(A)** Example of alignment grid points overlaid on a section image along with the original ROI (dashed red line) and ROI scaled by 110% (solid red line). Blue grid points are inside the area determined by the intersection of the scaled-ROI and a dilated version of the tissue mask. Red points are outside of this area. **(B)** Vector fields for neighbor comparisons of an example section. Blue points are inliers after the outlier detection, red points are outliers with their corresponding vectors removed, and gray points are points for which cross correlations were not computed [red points in **(A)**]. **(C)** Plot depicting the percentage of outliers for an entire dataset and averaged for neighbor comparisons in both directions at the specified neighbor distances. **(D)** Same vector fields in **(B)**, but with the points that were detected as outliers in **(B)**, the red points, and also the points at which no cross correlations were computed, the gray points, replaced with values estimated using the MLS rigid deformation algorithm. **(E)** Schematic showing two example sections for a single grid point with corresponding deltas and the vectors to be solved. **(F)** Matrices containing variables from the schematic in **(C)** that are solved by the fine alignment delta LSS which generates the fine (or ultrafine) alignment. **(G)** Distribution of solved deformation vector magnitudes from the fine and ultrafine delta LSS.

The 3D fine alignment proceeds after application of the rough alignment (global affine transformations per section) as essentially a constrained but fully elastic aligner. The nature of the deformations that occur during serial sectioning and section placement on the wafers can be local and non-linear, thus the rough alignment is not sufficient even for trained experts to trace neurites. There may still be misalignments present whose magnitudes are up to tens of microns. Therefore, we proceed with a finer alignment, which is accomplished in a straight forward manner, by computing a warping vector field that would be required for each section to warp it onto a neighboring (i ± n) section. The advantages of using comparisons beyond the direct neighbors in the section ordering are twofold: (1) any artifacts present in one or two sections can bridged without creating discontinuities in the alignment; (2) using more than just the direct section neighbors contributes to the “z-rigidity” of the alignment, meaning it helps to reduce, in particular, trend or low frequency oscillations in the final warping (as previously discussed in *3D Rough Alignment*). Multiple nxcorrs are performed between neighboring (i ± n) sections at fixed grid locations relative to the rough bounding box. It has been suggested that this approach fails if the raw EM data frequently contains discontinuities in the form of small folds or cracks (Macrina et al., 2021). We have solved this issue mostly on the experimental side (Fulton et al., 2023), such that we see a very low incidence of small folds or cracks in our raw EM micrographs. Template matching using nxcorr is performed on cropped regions from each image, a larger crop from the source image and a smaller crop from the destination image. The grid points are uniformly spaced (hexagonal layout) (Figure 8A). Tissue masks and acquisition ROIs can be optionally applied at this point, both as a method for saving compute time and also for reducing outlier cross correlations. If applied, cross correlations are only computed at locations within both scaled versions of the tissue mask and ROI (Figure 8A, blue dots). This step results in vector fields for all possible neighbor comparisons, where the vector at each grid location represents how far the source image would need to be translated in order to match the destination image at that grid point. Despite the ROIs and masks, outlier cross correlations are still possible, due to either a lack of structure (for example within cell bodies or blood vessels) or due to non-peaky topographies in the cross correlation images.

The procedure for removing outlier deltas in the 2D vector fields is composed of a series of heuristic steps that ultimately enforce 2D smoothness of the vector fields (Figure 8B). The principal step of the outlier detection utilizes RANSAC to fit the deltas to a polynomial affine model (typically second order), where outlier points from the best RANSAC fit are classified as outliers. The percentage of remaining inliers is a useful metric for detecting gaps or jumps between sections and also for assessing in general how many sections could be missed sequentially before leading to an alignment problem. This metric is simply the number of inliers deltas for a particular neighbor comparison divided by the number of grid points within the ROI and tissue mask. For an example dataset, the percentage drops to 40% and below when comparing the ±4 neighbors (Figure 8C). This also serves as a justification for not including further neighbors beyond four. Once the outliers are detected, they should be replaced with reasonable replacement x,y deltas. Without replacing outliers, the final alignment solutions can be very jumpy, particularly in areas where inlier deltas become very sparse. We replace all outliers by using the moving least squares (MLS) rigid image deformation algorithm (Schaefer et al., 2006), which is easily modified for interpolation / extrapolation of outlier deltas. The MLS algorithm results in realistic replacements for the outliers deltas (Figure 8D), and additionally, as opposed to classical interpolation, extrapolates points that are outside of the convex hull of inliers much more reasonably.

After 2D vector fields have had all outlier x,y deltas estimated, the complete vector fields are further constrained to enforce 2D smoothness and local rigidity, using a method that we termed an “affine filter.” The affine filter fits the x,y deltas in overlapping 2D windows with an affine transformation, and then replaces each center x,y delta with the one produced by the fit. In this sense it is similar to an image processing box filter, but filters vector fields and not images. This step is useful at reducing any unrealistic over-warpings that may occur at this stage in the alignment; since the crop size for the source images is typically chosen to be about 48 μm, any deltas of this size that are not consistent with neighboring deltas will produce extreme warpings that make the aligned data appear either extremely compressed or stretched. These smoothed vector fields are then reconciled independently per grid point using a fine alignment delta LSS (Figure 8E). This application of the fine alignment delta LSS (Figure 8F) is essentially identical to that of the rough alignment, except instead of a linear regression, a ridge regression may be utilized. Ridge regression includes an L2 regularization as another method of constraining the elastic alignment and prevent overwarpings. For the initial fine alignment (as opposed to subsequent refinements, i.e., the ultrafine alignment), the L2 regularization may not be necessary since the affine filtering has already constrained the vector fields to be locally smooth. The distribution of deformation vector magnitudes resulting from the fine and ultrafine alignment LSS (Figure 8G) demonstrates how well the *msemalign* package minimizes distortions. For the fine alignment of the zebrafish retina dataset the mean magnitude was 52.9 pixels (846 nm) and 99% of the magnitudes were below 133.6 pixels (2,138 nm). For the ultrafine alignment the mean magnitude was 7.4 pixels (118 nm) and 99% of the magnitudes were below 28.2 pixels (451 nm).

Once the fine alignment is completed, the *msemalign* pipeline currently requires that the images be exported, either at 4 nm, by simply scaling the transformations, or more typically again at 16 nm (~63 TBytes for a 1 PByte dataset). In what we term the ultrafine alignment, these images are then presented as input to the same *3D Fine Alignment* once again, but with different parameters. Primarily a much denser alignment grid and much smaller crop sizes in the source and destination images are chosen.

### Tear artifacts

**Figure 9:**
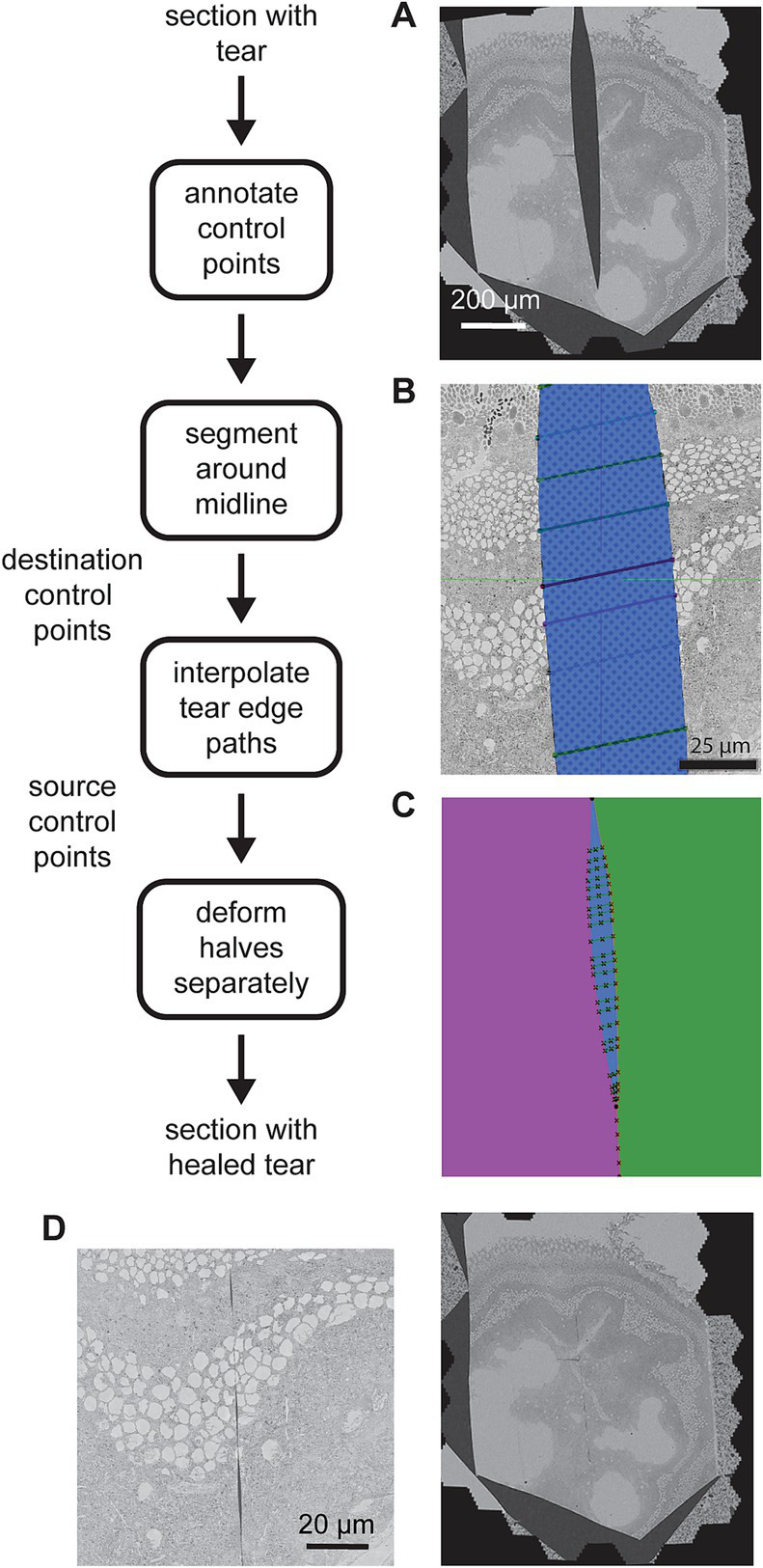
Detailed flowchart of the artifact correction steps. **(A)** Example section containing a large tear. **(B)** Illustration of the tear (blue) and control points (connected with line segments) in a zoomed portion of the same tear. **(C)** Result of segmenting the image area into halves (purple and green) on either side of the tear (blue). The manual control points and line segments connecting them are overlaid in black. The intersection of the line segments and the tear centerline are also marked (x’s along the tear centerline). **(D)** Same section as in **(A)** after the tear has been healed (left) and a zoomed view of the healed tear (right) showing some small gaps remaining at some locations between control points.

Although the issue of small cracks and folds has mostly been resolved with our acquisition method, there is still some occurrence of more serious discontinuities in the raw data that we have termed tears. Tears may occur when debris builds up on the diamond knife edge and this in turn can result in a section being cut into typically two pieces (Figure 9A). Many of the tears are clean, meaning there is no tissue missing at the tear boundary and the halves can simply be stitched back together. In order to avoid excluding these sections from the alignment, which may otherwise be in fine condition, we use a semi-automated method to stitch the halves together. In general, the 3D alignment assumes that the original sections are not badly deformed. Knife tears can open up to a width of 100 μm or more, meaning that torn sections are very deformed relative to their neighbors. Therefore we fix any tear artifacts directly after the 2D alignment (i.e., torn section images after the 2D alignment are replaced with the corrected ones), before the 3D alignment steps.

The manual portion of the tear correction involves identifying the torn sections and then creating annotations using webKnossos (Boergens et al., 2017). Annotations were performed by choosing paired corresponding control points on either side of the tear, resembling stitches (Figure 9B). The remainder of the correction is automated. First the midline of the tear is estimated by interpolating the center points of the annotated stiches. The midline skeleton is then projected (by fitting the ends) in order to create a path that divides the section into two pieces (Figure 9C, green and purple). The paired correspondence points are interpolated on either side of the tear independently and the interpolated points serve as source control points. The interpolated midline points serve as destination control points. The MLS algorithm is then employed to warp each half of the image separately on either side of the midline. Deforming each half separately prevents the MLS algorithm from dealing with the discontinuity where the two halves are pulled together. Because it assumes local affine rigidity, it would not handle such discontinuities well if the warping were to be applied to the whole section at once. When the halves are reassembled, the tear has been mostly healed (Figure 9D).

### Final stack export

**Figure 10:**
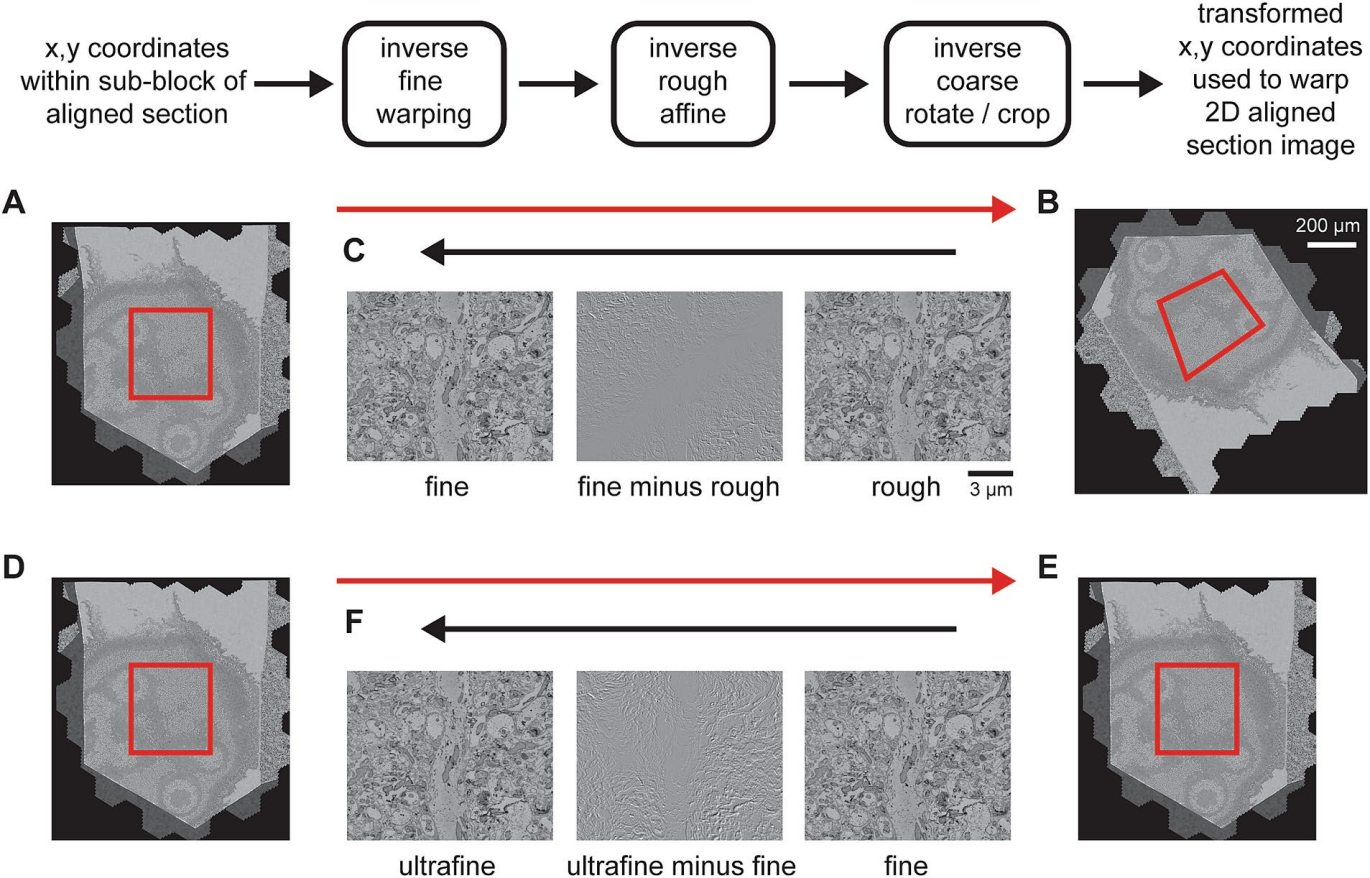
Detailed flowchart of the steps to export a final volume. **(A)** Section image of a temporary exported fine-aligned slice with an export block outlined in red. **(B)** 2D-aligned-only section image with an example of roughly how the block coordinates might be transformed (red outline). **(C)** A zoomed in area of the rough alignment (right) and the fine alignment (left) that have been centered on the same pixel. The difference between them (center) demonstrates what has changed between the rough and fine alignments. **(D–F)** Same as **(A–C)** but for the exporting the ultrafine based on an existing fine alignment export.

In order for the export to scale, particularly to the original 4 nm pixel size, the export of each section needs to be subdivided. Because the coarse and rough alignments are global per section and so that potentially multiple image transformations can be bundled as a single transformation, the export applies all transformations to pixel coordinates, starting with coordinates from a sub-block of the rough alignment bounding box. The rough bounding box represents a fixed reference frame, because, prior to the 3D alignment steps, all section images have been cropped to this size. The coordinates from a sub-block of the rough bounding box are then transformed with inverse transformations from each stage of the *msemalign* pipeline, but in the reverse order from which they were measured (Figures 10A–C). This procedure transforms the destination coordinates all the way back to the pixel space of the unoriented section images (the output of the 2D alignment), thus creating a destination to source image remapping. This remapping is then easily applied using general image warping tools available in many image processing libraries. As discussed, currently the *msemalign* pipeline runs the ultrafine alignment starting from an exported image stack of the fine alignment. Thus, after the ultrafine alignment is completed, this procedure is repeated, but only inverting the ultrafine transformations. The destination to source remapping is then applied using the fine alignment export images as the source (Figures 10D–F).

We applied the *msemalign* pipeline to a dataset of a larval zebrafish retina. The dataset was cut with 35 nm section thickness into 2,592 sections. A total of 19 sections were excluded due to section quality, so the 4 nm aligned dataset contains 78,750 × 75,344 × 2,573 voxels, or ~ 14 TB. The aligned volume can be viewed at https://webknossos.mpinb.mpg.de/links/4ig-0q1evJ649zfo.

## Discussion

The *msemalign* package and pipeline is an alignment solution for ssmSEM datasets that is scalable to petabyte-sized EM datasets and focuses on design simplicity. Relative to more complex alignment software ecosystems, *msemalign* has relatively few dependencies and can produce quality alignments using mostly traditional image processing approaches. Instead of setting a goal of complete generalizability to many alignment problems, we focused *msemalign* on constraints that are realistic to the sectioning and acquisition of ssmSEM datasets.

With sufficient hardware resources, for example, a modest cluster with on the order of 1000s of CPU cores and 100 s of GPUs, the *msemalign* pipeline can be scaled to process petabyte-scale datasets in approximately the same amount of time as the acquisition. With the exception of the order solving [only applicable if using the methodology of Fulton et al. (2023)], which scales with the square of the number of sections per wafer, most components simply scale linearly with the size of the dataset to be aligned. In the current design, the temporary downsampled versions of the dataset must be written out 3 times in the pixel size at which it is being processed, i.e., 16 nm (~63 TB for a 1 PB dataset acquired at a native 4 nm). These copies are: (1) the 2D aligned section stack, (2) the result of the fine alignment, and (3) the ultrafine alignment (the final export). The same temporary datasets must be written for the full 4 nm export, however these exports can be done with subsets of sections at a time, avoiding the heavy cost of writing multiple temporary petabyte-scale copies at full resolution.

The *msemalign* pipeline is under active development and further improvements are planned. In particular, we recognize the limitations in writing multiple, albeit temporary, downsampled copies of large datasets to disk. Having alignments proceed by necessitating downsampled copies to be written to disk allows for design simplicity, but limits the ability to ultimately scale to exabyte-sized datasets. It would be relatively straight forward to remove the requirement of writing out the fine alignment which serves as the input to the ultrafine alignment step. For each block of the ultrafine alignment cross correlations, the data could be loaded and transformed, via the export mechanism. Then cross correlations could be performed on cropped regions of this data in memory-only. There would be a relatively modest tradeoff for compute time in applying the transformations of the fine alignment. The final export would have to apply two inverse warpings to the coordinates, first the ultrafine inverse warpings and then the fine inverse warpings. Removing the requirement to write out the 2D aligned stack, such that the data needed for the 3D alignment is always loaded from the raw mSEM tiles, would introduce further complexity to the pipeline, mostly due to the feathering between images that is applied during the 2D montaging. The feathering utilizes ramps that are generated in 2D based on the final overlaps between images, which are only calculated after the images are overlaid at their solved coordinates. Storing a lookup of the feathering parameters would however be possible and would remove the current requirement of writing the 2D aligned stack to disk. On the other hand, we routinely utilize the temporary downsampled versions of the dataset as quality controls of a particular dataset as it passes through the pipeline to identify any issues and tune parameters as needed.

A relatively simple enhancement to the rough alignment would be to compute image features using multiple algorithms (in addition to SIFT), match them separately and then pool them together. Different feature detection algorithms together can likely increase the number of matching keypoints, particularly in situations where multiple sequential sections are missing or between images that appear different due to section thickness variation.

The 3D contrast and brightness correction could be enhanced by using multiple template histograms from differently sampled points in the section ordering. Conventional histogram equalization uses a uniform grayscale histogram as the target histogram. This type of normalization, or with a localized adaptation such as CLAHE, is a more conventional method of contrast balancing EM data. These procedures, however, tend to equalize many actual tissue structure differences, leading to regions appearing more similar than they may appear in the original micrographs (for example collections of cell bodies versus neuropil, or ganglion cell bodies versus photoreceptors in the retina). Our approach of histogram matching to a template prevents this artifact of equalization and retains the look and feel of the original EM micrographs. However, for large z-stacks, assuming a constant histogram target is unrealistic. A possible enhancement would be to sample various high contrast sections along the section stack. These histograms could be smoothly resampled, so that a series of target histograms as a function of the section z-coordinate is created. This would further maintain something closer to the original grayscale distributions of the EM micrographs, but still balance and correct lower contrast sections.

## Data availability statement

The original contributions presented in the study are included in the article/supplementary material, further inquiries can be directed to the corresponding author.

## Ethics statement

The animal study was approved by Landesamt für Natur, Umwelt und Verbraucherschutz Nordrhein-Westfalen, Germany. The study was conducted in accordance with the local legislation and institutional requirements.

## Author contributions

PW: Conceptualization, Formal analysis, Methodology, Software, Writing – original draft, Writing – review & editing. EJ: Methodology, Software, Writing – review & editing. KB: Conceptualization, Funding acquisition, Resources, Supervision, Writing – original draft, Writing – review & editing.

## Footnotes

^1^ https://webknossos.mpinb.mpg.de/links/4ig-0q1evJ649zfo

^2^ https://github.com/mpinb/msemalign

^3^ https://github.com/mpinb/swarm-scripts

^4^ https://github.com/mpinb/rcc-xcorr
